# Nutritional status of Palestinian preschoolers in the Gaza Strip: a cross-sectional study

**DOI:** 10.1186/1471-2458-12-27

**Published:** 2012-01-11

**Authors:** Salwa G Massad, FJ Nieto, Mari Palta, Maureen Smith, Roseanne Clark, Abdel-Aziz Thabet

**Affiliations:** 1 Department of Economics, BirZeit University, BirZeit, Palestinian Territory; 2 Department of Population Health Sciences, University of Wisconsin-Madison, Madison, USA; 3 Department of Psychiatry, University of Wisconsin-Madison, Madison, USA; 4 Child Institute, Al Quds University -Gaza Branch, Gaza Strip, Palestinian Territory

## Abstract

**Background:**

The authors examined factors associated with nutritional resilience/vulnerability among preschoolers in the Gaza Strip in 2007, where political violence and deprivation are widespread.

**Methods:**

This cross-sectional study was carried out in 2007 using random sampling of kindergartens in order to select 350 preschoolers. Binary logistic regression was used to compare resilient (adequate nutrition) and vulnerable (stunted) groups with those with moderate nutrition.

**Results:**

Approximately 37% of the subjects demonstrated nutritional resilience and 15% were vulnerable. Factors associated with nutritional resilience were child younger age, normal birth weight, actively hand- or spoon-feeding when the child was below two years, and residential stability in the past two years. The only factor associated with nutritional vulnerability was lower total score on the mother's General Health Questionnaire, which we interpret as a marker of maternal mental health.

**Conclusions:**

Children with low-birth weight and older children had worse nutritional resiliency outcomes. Further, poorer outcomes for children were associated with lower maternal mental health status, as well as increased family residential instability. Our results add to the large literature on the pervasive effects of violence and instability on children and underscore the need for resources for early intervention and for the urgent resolution of the Palestinian and other armed conflicts.

## Background

The total population of the Palestinian Territory^a ^by the end of 2010 was 4.1 million; 2.5 million in the West Bank and 1.6 million in the Gaza Strip. About 43.7% of Palestinians are refugees, 29.3% in the West Bank and 67.5% in the Gaza Strip [1]. In September of 2000, political violence resulted in the imposition of restrictions on the movement of Palestinian goods and people across the borders and within the Palestinian Territory. These restrictions seriously compromised household welfare resulting in loss of income, decreased quantity and quality of food, and impeded access to health care [2-5]. The extremely challenging living conditions in the Gaza Strip are well known [6,7]. In 2003, a study showed that the prevalence of anemia among children under 2 years old in Gaza was 72.8%, while the prevalence of wasting, stunting, and underweight was 34.3%, 31.4%, 31.45% respectively [8]. Poverty rates in 2007 were 51.8% in Gaza and 19.1% in West Bank; when food aid and remittances were excluded, the rates rose to 79.4% in Gaza and 45.7% in West Bank [9]. Stunting of children under 5 has increased by 41.3% since the Uprising in 2000 when it was reported at 7.5% [1]. In 2010, 19.4% of children aged 6-59 months suffered from anemia; 25.6% in Gaza and 13.4% in West Bank [1]. However, the nutritional consequences for children have received little attention. Previous local nutrition studies among school children [10-14], pregnant women [15], and those among preschoolers [16-20] had exclusive focus on nutritional vulnerability, which is the case with nutrition research in general, i.e., the study of determinants of malnutrition only [21-29].

The study of resilient children acknowledges that the determinants of resilience and vulnerability might be different, based on the theory that the mechanisms through which they operate differ [30]. Resilience is the individual's ability to resist the potential negative consequences of the risk factors and develop adequately [31]. Risk factors include individual-level characteristics such as low birth weight; household-level factors such as family poverty; and community-level factors such as living in a community that has suffered from political violence. Most studies of nutritional resilience in developing countries have focused on children's feeding, hygiene, maternal education, employment, and income, while little is known about the effect of maternal mental health and deprivation [21-23,32-40].

Anthropometric assessment (height-for-age, weight-for-age, and weight-for-height) is widely used and often regarded as the best single measure for health and nutritional status in children [41]. Malnutrition, mostly in its mild or moderate forms, contributes to more than half of the eleven million deaths each year among children under 5 years of age in developing countries, and diminishes capacities among those who survive [41]. Chronic malnutrition is commonly measured by stunting, which is defined as low height-for-age [41-44]. The negative consequences of stunting include lower learning and work capacity and higher risk of morbidity and mortality from birth through childhood. Among girls, it increases the risk of obstructed labor and thereby of maternal mortality [45].

The causes of malnutrition are complex and multifaceted. In developing countries; dietary factors (intake of dietary supplements (iron and vitamin A and D) [46], exclusive breast feeding for 4-6 months, complementary feeding at 6 months [47]), maternal education, maternal mental health [48,49], and family socioeconomic and environmental factors (deprivation, social support, and hygiene [37]) all may be associated with malnutrition [26,27,50-52]. In addition, maternal mental health has been shown to suffer with exposure to war-related violence [48,53].

We sought to describe the variability of nutritional status and its association with various child, socioeconomic and maternal factors in preschoolers, who comprise 18% of the Gaza Strip population [54]. We postulated that larger size of the household, war exposure, economic deprivation, low birth weight [29,55], poor maternal mental health [48,49], poor maternal self- rated health, and low education [29,55] might be associated with nutritional vulnerability, while actively feeding but not forcing the child to eat [21], social support, and residential stability in the past two years are potentially related to nutritional resilience. Furthermore, we postulate that there will be significant variability in vulnerability and resilience by locality due to variability in deprivation and war exposure.

## Methods

### Description of target population and sampling design

In the Gaza Strip, the region most adversely affected by the political conflict and deprivation in Palestine, we conducted a cross-sectional kindergarten-based survey of preschoolers. Although the kindergartens are licensed for children 4 to 5 years old, there were some 3 and 6 year olds in the selected kindergartens. The average household size in Gaza is 7.0 people and the fertility rate is 5.4 births per woman [54].

As previously described [53], we randomly selected ten kindergartens across the Gaza Strip. The random sample of kindergartens included four cities, two villages, and two camps. Six of the randomly selected kindergartens were from Gaza city, Der El Balah city, Rafah and Khan Younis cities. Two were selected from Beach camp and Nusirate camp, and two from Beit Hanoun and Zwaida villages. These represent the different types of kindergartens found in Gaza, two are United Nations Relief and Works Agency for Palestine Refugees in the Near East (UNRWA), two public, and six are private. All preschoolers 3-6 years old were selected from the registration books of the ten kindergartens. We randomly selected only one child from each family. If a family had more than one child in the same age group, selection proceeded as follows. The youngest was selected in the first family, the second youngest in the second family, the second oldest child in the third family, and the eldest in the fourth family was approached. This pattern was repeated in subsequent families. Acting as our Institutional Review Board (IRB), the Human Subjects Office, College of Letters and Sciences at University of Wisconsin-Madison, approved the study protocol in December 2006.

### Recruitment

The field manager called parents using phone numbers in registration books, briefed them about the study, and invited the mothers to come to the kindergartens to participate. All selected families were contacted through either cell phones or landlines. We found that at the time this study was conducted, most mothers did not feel comfortable with signing papers, thus making the standard written research consent form culturally inappropriate in this Palestinian population. Thus, in consultation with our IRB, verbal informed consent was obtained from the mothers following a description of the study. The mothers were given the option to skip any question they did not feel comfortable answering, and to temporarily or permanently stop the interview. The response rate was 100%. This high response rate was expected as a previous cross-sectional survey and Palestinian Demographic Health Surveys (2000, 2002, 2004, and 2006) had response rates between 95.5 and 98.3% [54,56-59].

### Nutritional status assessment

The main outcomes of interest were nutritional resilience and nutritional vulnerability. Standing height of each child was measured following World Health Organization recommendations [60]. For children up to 5 years old, we used "WHO ANTHRO 2005" program in the analysis of the anthropometric data (height-for-age) [61]. Since "WHO ANTHRO 2005" does not cover children older than 5 years, we used "NutriSurvey for Windows" for 6 year-olds for score standardization [62]. The two software packages are comparable, as both use 1977 National Center for Health Statistics (NCHS)/WHO reference population [63]. We used Z-scores for height-for-age of less than -2.0 as the cut-off point for nutritional vulnerability [41,60]. We defined moderate nutrition as Z-scores between -2.0 and 0.0 and nutritional resilience as a Z-score 0.0 or above [21]. For example, a 5 year-old girl would be considered vulnerable if her height was 98.0 cm, while the same girl would be considered having moderate nutrition or resilient if her height was 104.0 or 110.0 cm.

### Explanatory variables

The main child level explanatory variables were: age, gender, birth order, birth weight (based on mother's report), positive feeding practices (active feeding the child and not forcing the child to eat), intake of dietary supplements (iron and vitamin A and D) [46], exclusive breast feeding for 4-6 months, and complementary feeding at 6 months [47]. The maternal factors were: mental health, self-rated health, and education. The household factors were: family size, number of rooms in the house, hygiene, residential stability in the past two years, living in direct military confrontation area, locality (some are the most disadvantaged among the eight localities in terms of political violence and deprivation), economic deprivation, and social support as defined below.

### Variable definitions

#### Active feeding

Actively hand or spoon feeding the child when he was below two years of age. Measured by asking the mother whether she actively fed her child when she or he was below 2 years of age.

#### Absence of forced feeding

This variable was measured by asking the mother whether she forces her child to eat in the event he/she refuses to eat.

#### Low birth weight

Birth weight < 2.5 kg. This variable was measured based on mother's report.

#### Hygiene

This rating was constructed by summing the responses (Yes = 1, No = 0) to the following dichotomous items observed by the interviewer: mother's nails and clothes were clean and child's nails and clothes were clean [37].

#### Deprivation

We constructed a deprivation score by adding the number of affirmative responses to the three questions about not having enough money for living expenses, not having enough money to pay bills, and the mother feeling that her child was deprived [52,54]. In addition to the summative score deprivation variable, we present descriptive statistics in Table 1 about access to meat, fruits and vegetables, having sufficient quantity of food, and being on food assistance.

**Table 1:** Characteristics of the study sample (N = 350), Gaza, 2007

	N (%) (unless otherwise indicated)
Child factors	

Age in months [mean (SD)]	59 (8)

Females	170 (49%)

Low birth weight (< 2.5 Kg)	25 (7%)

Received iron syrup	209 (60%)

Received vitamins A &D	197 (56%)

Exclusively breast fed for 4-6 months	278 (79%)

Complementary food at 6 months	237 (68%)

Resilient^1^	130 (37%)

Vulnerable^2^	52 (15%)

Moderate^3^	168 (48%)

Maternal factors	

Age in months [mean (SD)]	31 (6)

Elementary Schooling only	34 (10%)

Employed	24 (7%)

Perceived health	

- Excellent	45 (13%)

- Very good	82 (23%)

- Good	111 (32%)

- Fair or poor	112 (32%)

Potential mental health problem^4^	209 (60%)

Household factors	

Living in a direct military confrontation area	88 (25%)

Family moved in the past 2 years	65 (19%)

Average household income in US dollars^5 ^[mean (SD)]	204 (174)

No meat/chicken/fish most days of week^6^	197 (56%)

No fruits and vegetables most days of week^6^	212 (61%)

Family did not have enough food for at least 2 days in the past month	146 (42%)

On food assistance^6^	176 (50%)

Deprivation^6^	

Shortage of money for living expenses	42 (12%)

No money to pay bills	63 (18%)

Mother thought her child was deprived	55 (16%)

Reported any form of social support^7^	132 (38%)

Family size [mean (SD)]	8 (4)

Number of rooms in the house [mean (SD)]^8^	3 (1)

*Source of data: *Interviews with mothers of 350 children aged 3-6 in 10 kindergartens across Gaza Strip, Palestine, January 2007.

^1 ^Height-for-age Z-score ≥ 0 (adequately nourished, at or above reference population mean).

^2 ^Height-for-age Z-score < -2 (stunted, more than 2SD below the reference population mean).

^3 ^-2 ≤ Height-for-age Z-score < 0 (in between group, moderate nutritional status, up to 2SD below the reference population mean, still within the normal range)

^4 ^Total score on General Health Questionnaire ≥ 5.

^5 ^The estimate is based on 120 households.

^6 ^Three months prior the survey.

^7 ^The mother had someone to trust, to count on for help, had someone with whom she feels intimacy, or had family and friends that make her happy and secure.

^8 ^Excluding bathroom and kitchen.

#### Confrontation level

Measured by asking the mothers if their dwelling lies in a direct military confrontation area, where fighting between Israeli soldiers and the Palestinians and military operations are frequent (Yes/No).

#### Residential stability

Measured by asking the mother if the family moved in the past 2 years.

#### Social support

The perceived social support factor was drawn from the items on the Social Provisions Scale [64]. Rating was based on the yes/no answers to the following items: mother has someone to count on for help, has friends and family to make her happy and secure, has somebody to trust to talk about problems, and has someone with whom she feels intimacy [65]. In our sample, Cronbach's alpha (a reliability measure capturing the internal consistency by the intercorrelation of items) for the Social Support Scale was 0.75, which is greater than 0.70, the standard recommended for group comparison (with values closer to 1 more reliable).

#### Maternal mental health

A rating was based on responses to the General Health Questionnaire (GHQ-28) [66]. The GHQ-28 covers severe depression and suicide risk, anxiety and insomnia, social dysfunction, and somatic symptoms [66]. GHQ-28 scores above the cutoff of 4/5 are considered to identify possible psychiatric 'cases' [67]. A validation study of the GHQ-28, in comparison with the Clinical Interview Schedule, yielded a sensitivity and specificity to diagnose possible psychiatric cases of 88.0% and 84.2%, respectively [66]. In a previous study, the Cronbach's alpha for the GHQ-28 was 0.91 and test-retest coefficient after six months was 0.90 [66]. In our sample, the Cronbach's alpha for GHQ-28 was 0.87.

#### Maternal self-rated health

We measured health perceptions through the question, "At the present time, would you say your health is excellent, very good, good, fair, or poor?" We coded this variable into a binary variable: fair or poor versus good/very good/or excellent.

We translated the study questionnaire into Arabic and then translated it back to English. Subsequently, it was pilot-tested among mothers of 35 preschoolers in the Gaza Strip. Finally, we revised the questionnaire and administered it to the study sample following written guidelines.

### Statistical analysis

We analyzed our dataset of 350 subjects with SPSS 14.0 (Statistical Package for the Social Sciences). We used means and percentages to describe the characteristics of the study sample. In addition to estimating the overall prevalence of nutritional resilience and vulnerability, we calculated the prevalence by locality (8 localities). Chi-square test and analysis of variance (ANOVA) were performed to examine differences in socioeconomic status between the resilient, moderate, and vulnerable groups and differences in nutritional status and adversity (living in a direct military confrontation area and family did not have enough food for at least two days in the past month) by locality. We used binary logistic regression analysis to examine child, mother, war exposure and household factors associated with resilience and vulnerability, using those with moderate nutrition as a reference group [21,68]. The final models included all variables with a p-value ≤0.05. Tests of significance were two-sided. Four models each were constructed for resilience and vulnerability. Model 1 included child factors (age, gender, birth order, birth weight, positive feeding practices (actively feeding the child and not forcing the child to eat), intake of dietary supplements (iron and vitamin A and D) [46], exclusive breast feeding for 4-6 months, complementary feeding at 6 months); Model 2 included child factor and war exposure (residential stability, living in a direct military confrontation area); Model 3 included child factors, war exposure, and household factors (deprivation, social support, hygiene, number of rooms in the house, and family size); and Model 4 included child factors, war exposure, household factors, and maternal factors (mental health, self-rated health, and education).

## Results

### Sample characteristics

Of the children studied, 49% were female (N = 170) (Table 1). Based on mothers' reports, the children's birth weight ranged from 1.2 to 5.0 kg, almost 7% of the children had low birth weight. Approximately 37% (N = 130) of the subjects demonstrated nutritional resilience and 15% (N = 52) were vulnerable. While the geographical variability in nutritional resilience (22-53%) was insignificant (*p *= 0.08), nutritional vulnerability (3-27%) varied significantly by locality (*p *= 0.03) (Table 2). Living in a direct military confrontation area, the family did not have enough food for at least two days in the past month, and receiving food assistance on regular basis varied significantly by locality. Mean maternal age was 31 years, and 10% had elementary schooling or less (i.e. a 6th grade or lower level of education). Almost 32% of the mothers rated their health as fair or poor, and 60% had potential mental health problems based on the GHQ-28. More than half of the study participants had no money to pay bills, had no meat, fish, fruits, or vegetables most days of the week, or were on food assistance (Table 1). As shown in Table 3, the resilient, moderate, and vulnerable groups are comparable in terms of maternal education and employment and household factors: deprivation, average monthly income, family did not have enough food for at least two days in the past month, being on food assistance, size of the household, number of rooms in the house, and living in direct military confrontation area. Therefore, children labeled as resilient came from the same environment as those labeled as vulnerable; they experienced the same conditions yet have a different outcome.

**Table 2:** Prevalence of nutritional resilience and vulnerability by locality, Gaza 2007 (N = 350)

	Beit Hanoun (N = 35)	Beach Camp (N = 37)	Gaza City (N = 68)	Nusirat Camp (N = 34)	Zwaida village (N = 36)	Deir Al lbalah (N = 70)	Khan Yunis (N = 36)	Rafah City (N = 34)	p-value^1^
Living in a direct military confrontation area	94.3%	0%	17.7%	14.7%	2.8%	12.9%	33.3%	47.1%	**0.00**

Family did not have enough food for at least 2 days in the past month	65.7%	70.3%	25.0%	35.3%	13.9%	38.6%	38.9%	64.7%	**0.00**

Family received food assistance on regular basis	91.4%	24.3%	54.4%	58.8%	30.6%	42.9%	50.0%	60.6%	**0.00**

Nutritional vulnerability^2 ^(Stunting)	8.6%	27%	23.5%	8.8%	2.8%	14.3%	16.7%	8.8%	**0.03**

Nutritional resilient^3 ^(Adequate nutrition)	28.6%	21.6%	32.4%	44.1%	52.8%	44.3%	30.6%	41.2%	0.08

*Source of data: *Interviews with mothers of 350 children aged 3-6 in 10 kindergartens across Gaza Strip, Palestine, January 2007.

^1^Based on Chi square test

^2 ^Height-for-age Z-score < -2 (stunted).

^3^Height-for-age Z-score ≥ 0 (adequately nourished).

**Table 3:** Mean differences between resilient, moderate, and vulnerable groups in socioeconomic status and exposure to political violence, Gaza 2007

	Resilient (N = 130)	Moderate (N = 168)	Vulnerable (N = 52)	All (N = 350)
Maternal factors				
Elementary Schooling only	8.5%	8.3%	17.3%	9.7%

Employed	4.6%	8.9%	5.8%	6.9%

Household factors				

Living in a direct military confrontation area	26.1%	25.0%	23.1%	25.1%

On food assistance	50.0%	49.7%	52.0%	50.7%

Family did not have enough food for at least 2 days in the past month	40.0%	43.5%	40.4%	41.7%

Deprivation score^1 ^(mean [SD])	1.3 [1.2]	1.5 [1.4]	1.6 [1.4]	1.4[1.3]

Average household income in US^2 ^dollars^4^(mean [SD])	205 [134]	206 [190]	197 [200]	204 [174]

Family size (mean [SD])	7.9 [3.9]	8.0 [3.8]	9.1 [4.4]	8.1 [3.9]

Number of rooms (mean [SD])	3.1 [1.3]	3.0 [1.0]	3.1 [1.1]	3.1 [1.1]

*Source of data: *Interviews with mothers of 350 children aged 3-6 in 10 kindergartens across Gaza Strip, Palestine, January 2007.

Note: Based on Anova test and chi square test, none of the mean group differences were statistically significant at *p *≤ 0.05

^1: ^A rating was constructed as the number of affirmative responses to the following dichotomous items: family did not have enough money for living expenses, did not have money to pay the bills, and the mother felt that her child was deprived.

^2: ^Only 31% and 29% of families of vulnerable and resilient children reported income, respectively.

### Factors associated with nutritional resilience and vulnerability

#### Factors associated with nutritional resilience (Table 4)

The factors that emerged as significantly associated with nutritional resilience were the age of the child (odds ratio [OR] per one month increment = 0.95, 95% confidence interval: 0.92, 0.98), normal birth weight (≥ 2.5 Kg) (OR = 5.85, 95% CI: 1.64, 20.94), actively hand- or spoon-feeding when the child was below 2 years of age (OR = 3.23, 95% CI: 1.32, 7.69), and residential stability in the past two years (OR = 1.98, 95% CI: 1.04, 3.77). Child gender, birth order, child dietary supplements, timely introduction of complementary food (at 6 months), exclusive breastfeeding for 4-6 months, hygiene, and social support were not associated with nutritional resilience (all p-value > 0.05; results not shown).

**Table 4:** Factors associated with nutritional resilience among preschoolers,^1 ^Gaza, 2007

	Resilience	
**Variable**	**Odds Ratio**	**95% Confidence Interval**

Age (per month)	0.95	0.92, 0.98

Normal birth weight	5.85	1.64, 20.94

"Mother helped feeding the child when he was younger than 2 years"	3.23	1.32, 7.69

Residential stability in past 2 years	1.98	1.04, 3.77

*Source of data: *Interviews with mothers of 350 children aged 3-6 in 10 kindergartens across Gaza Strip, Palestine, January 2007.

^1^: Based on Binary logistic regression, with the moderate nutritional status as a referent group. Only variables with regression coefficients associated with a p-value ≤ 0.05 were included in the model.

#### Factors associated with nutritional vulnerability

Based on logistic regression (Table 5), the only factor associated with nutritional vulnerability was mother's lower total mental health score on GHQ-28 (OR = 1.07, 95% CI: 1.01, 1.13). Adjusting for gender, child dietary supplements, timely introduction of complementary food, exclusive breastfeeding for 4-6 months, and deprivation, did not substantively change the magnitude or significance of the associations. Larger size of the household, living in direct military confrontation area, economic deprivation, poor maternal self-rated health, low birth weight, higher birth order, fair or poor quality of life, and low education were not associated with nutritional vulnerability (not shown).

**Table 5:** Factors associated with nutritional vulnerability^1^, Gaza, 2007

	Vulnerability	
**Variable**	**Odds ratio**	**95% Confidence Interval**

Total score on GHQ-28^2^	1.07	1.01, 1.13

*Source of data: *Interviews with mothers of 350 children aged 3-6 in 10 kindergartens across Gaza Strip, Palestine, January 2007

^1^: Based on Binary logistic regression, with the moderate nutritional status as a referent group. GHQ score was the only variable with a regression coefficient associated with a p-value ≤ 0.05.

## Discussion

Malnutrition is a serious public health problem in the Gaza Strip. We examined both resilient and vulnerable groups to understand how families succeed and how they fail in supporting child nutrition in the face of adversity. This study adds to the body of literature about malnutrition in Palestine by demonstrating how poor maternal mental health, low birth weight, and residential instability contribute to nutritional vulnerability among kindergarten-aged children. It also suggests that children who are assisted with eating by their mothers before the age of 2 are three times more likely to be nutritionally resilient, and that older children are significantly less resilient than younger ones.

In agreement with previous studies undertaken in the Philippines and Tanzania [69,70], low birth weight children are less likely to show nutritional resilience than those with normal birth weight and equally more likely to show vulnerability, although the later association was not statistically significant, possibly due to the smaller number of vulnerable children (53 children with stunting). Based on the study sample, older children are less likely to show resilience, which is in line with our previous finding that older children had a poorer quality of life [71]. This may be explained by more exposures to traumatic events among older children which might have a cumulative effect [72].

Based on study findings, child gender is not associated with nutritional status among preschoolers, which is in line with a previous study in Gaza that showed no gender differences in nutritional status or feeding patterns among infants below 18 months of age [73]. Maternal low level of education is not associated with nutritional vulnerability, a finding that contradicts previous studies in China [29,55], Indonesia [36], Bangladesh [74], India [26], Nepal [27], and Thailand [24]. Poor maternal mental health is associated with nutritional vulnerability, a finding in agreement with other studies in India and Pakistan that found maternal postpartum depression to be associated with malnutrition in children [48,49]. Similarly, a study in Brazil found that poor maternal mental health was associated with a threefold higher risk of nutritional vulnerability [75]. Another study in rural Chad showed that maternal psychosocial characteristics were among the best predictors of child height-for-age [76]. Poor maternal involvement in childcare may be one of the explanations for the association between maternal depression and child malnutrition [77,78]. Depressed mothers are more likely to be withdrawn, passive, and less responsive to their children, as well as less able to establish and maintain positive interactions [77,78]. In addition, higher scores on maternal depression have been found to be associated with persistent food refusal behavior by the child [79]. However, in this study, the relation between maternal mental health and child nutritional vulnerability was not explained by feeding behaviors including force feeding and helping feed the child when he or she was below 2 years of age, as both variables did not affect the significance of the association between maternal mental health and nutritional vulnerability (data not shown). The association between poor maternal mental health and nutritional vulnerability may also be in part explained by reverse causation, i.e., mothers may have poor mental health because their children are undernourished. However, an evaluation of a five-month psychosocial intervention program for mothers in war-torn Bosnia and Herzegovina showed positive effects on children's weight [80]. Another study among 221 infants found that infants of mothers with depressive symptoms experience poor linear growth when followed for six months [81].

We did not find an association between the absence of forced feeding of the child and nutritional resilience, as had been reported by earlier studies [21,37,82,83]. This difference in findings may be because food is not always available in the Gaza Strip, thus food refusal may be less of an issue. Following the Uprising in 2000, loss of jobs, assets and incomes sharply reduced economic access to food with real per capita income decreasing by half since 1999 and resulting in six out of ten people falling below the US$ 2.10 per day poverty line in mid-2006 [84]. We did not find an association between living in direct military confrontation areas and malnutrition. This is in line with the findings of our previous study of health related quality of life of preschoolers where living in direct military confrontation areas was associated with psychosocial health but not with physical health [71]. However, the study highlights the association between residential instability in the Gaza Strip and the lower prevalence of nutritional resilience.

Contrary to previous studies in developing countries, [22,85-87] our data did not support an association between intake of supplements, birth order, exclusive breast feeding, timely introduction of complementary food, size of the household, and/or economic deprivation and the child's nutritional status. One possible explanation is that other factors such as maternal mental health may be more critical to children's growth when exposed to adversity. Lack of association between intake of dietary supplements and child nutritional status, may be due to reverse causation, as malnourished children were more likely to take dietary supplements when they were younger. In contrast to our expectations, mother's social support was not associated with child nutritional status. This is consistent with our previous study where social support was not associated with child physical health [71]. This may be explained by the high prevalence of poor mental health among the mothers (60% according to our study results) and thereby the inability to offer or make use of support to help buffer the negative effects of war. Another possible explanation is, again, reverse causation, as children/families with nutritional deficiencies might attract more sympathy from their neighbors/relatives.

We believe that our estimate of 15% vulnerable children may be an underestimate of the true prevalence of malnutrition in the Gaza Strip, as only one third of children 3-6 years old go to kindergarten. Those who fail to enroll do so most often because their families cannot afford it, or because they live in violent areas and their families are worried about their safety [88,89]. However, our results are similar to those obtained by the Palestinian Demographic Health Survey (DHS 2006) (data collected Nov 2006-January 2007). In this survey, 4673 children under five were examined in the Gaza Strip yielding a prevalence of vulnerability of 13% [54].

In line with earlier findings that some children show nutritional resilience despite adversity [21,90], 37% of the children in our study were nutritionally resilient. Although there was insignificant variability in nutritional resilience by locality, there was significant variability in nutritional vulnerability. Beach camp had the highest prevalence of nutritional vulnerability and the lowest prevalence of resilience, while the opposite was noticed in Zwaida village. Beach camp is a refugee camp in Gaza city with high level of unemployment and overcrowding of families in a very small area, with a large number of children [91]. While Zwaida village is in the middle area of the Gaza Strip and most of the families are landlords and they cultivate their lands and have more income and larger houses than families in the nearby refugee camps. This is evident from the difference in reporting family insufficient food for at least two days in the past month, 70% vs. 14%, respectively. Almost 94% of residents of Beit Hanoun reported living in a direct military confrontation area as this town is only a few hundred metres from the Palestinian-Israeli border and has a high level of confrontation. The relatively low prevalence of nutritional vulnerability in Beit Hanoun can be explained by the highest percentage of households in Beit Hanoun receiving food assistance on a regular basis (at the time of the study) compared to the other localities.

We examined both resilient and vulnerable children to address the causes of malnutrition. Timely interventions may prevent and reverse malnutrition among preschoolers [92]. Given the results of this study, interventions should target maternal mental health and residential stability. In a previous study, poor maternal mental health was associated with living in a direct military confrontation area [53]. It is hard to target residential instability and poor maternal mental health by interventions short of ending the violence. Consequently, addressing the root cause of nutritional variability might require the ending of the political violence. Further confirmatory studies should be conducted that include children from the West Bank to see if associations hold, and to examine the effect of the political changes, and the increased intensity of violence in the Gaza Strip on children's nutritional status. We also recommend replicating the study among other deprived populations, and those exposed to domestic and neighborhood violence, to see if the findings are consistent with our findings in the context of political violence.

The cross-sectional design of the study precludes examination of causality. As malnutrition is one of the main factors contributing to child mortality [93], our estimate of the prevalence of nutritional vulnerability might have been low compared to the true prevalence at a younger age, due to survival bias. Similarly, the estimate of resilience may be overestimated in our study. Another possible limitation of our study is that, as it is not culturally acceptable in Palestine, we were unable to measure mother's weight and height to control for maternal nutritional status, a variable that has been associated with the risk of childhood malnutrition in other studies [43]. Other limitations are that child birth weight was based on mother's report, and the small number of vulnerable children (53). The latter affected the power to detect significant associations. Finally, the lack of observational or quantitative detail on some of the information collected (e.g., feeding style or habits) limited our ability for more specific and objective analyses.

This study is unique in several respects. First, it examined both nutritional resilience and vulnerability of preschoolers exposed to political violence and deprivation in particular. Second, it accounted for maternal mental health and social support in studying children's nutritional status. Third, it assessed the effect of several risk factors simultaneously, which is important in identifying the combinations of factors that increase nutritional vulnerability of children. Fourth, the study context provided a unique opportunity to examine the impact of both chronic and acute exposure to political violence on children's health. Preschoolers in our study were born and raised following the Uprising in 2000. Since 2006, there has been an escalation in the conflict in the Gaza Strip, compounding with an increase in Palestinian internal violence.

## Conclusions

Our study shows that children exposed to political violence have serious health issues and underscores the need for early preventive intervention and for the urgent resolution of the Palestinian and other armed conflicts. The study identified modifiable risk factors that can promote resilience and reduce vulnerability in children exposed to political violence and deprivation. The high prevalence of both child nutritional vulnerability and poor maternal mental health (and its strong association with malnutrition), call for psychosocial interventions that can have positive effects on the mental health of the mothers as well as the nutritional status of their children. Such interventions should address the mother's emotional well-being and provide coping strategies as well as education and counseling to promote the development and well-being of the children through parental involvement and support. There is also a need for prenatal interventions to identify mothers at risk of delivering a preterm or growth-retarded infant and to provide medical, nutritional, and educational interventions intended to reduce the determinants and incidence of low birth weight. The study also highlights the importance of promoting good feeding practices to promote nutritional resilience. It is hoped that these findings will assist in the revision of child and family oriented programs and policies in areas of armed conflict, in order to reduce mortality and morbidity in children and contribute to their well-being. It is not too late to intervene, as a longitudinal study among 1674 children found no significant difference between early (stunted at 6-18 months of age) and concurrent stunting (stunted at 4.5-6 years of age) on cognitive ability. This suggests that preventing stunting should not only focus on the under 2 s but include children up to 5 years of age [94].

## Endnotes

^a^The Palestinian Territories consist of two distinct areas: the Gaza Strip, the West Bank. The eastern limit of the West Bank is the border with Jordan, while the southern limit of the Gaza Strip is the border with Egypt.

## Abbreviations

UNRWA: United Nations Relief and Works Agency for Palestine Refugees in the Near East; GHQ: General Health Questionnaire.

## Competing interests

The authors declare that they have no competing interests.

## Authors' contributions

SGM made contributions to the study design, data collection, statistical analysis, interpretation of data and the drafting of the manuscript. FJN contributed to the study design, statistical analysis, interpretation of data and drafting of the manuscript. MP contributed to the study design, guided the statistical analysis and contributed to the interpretation of results and to writing and editing the manuscript. MS contributed to the study design, data collection, interpretation of data, and manuscript writing and editing. RC contributed to interpretation of data, and manuscript writing and editing. AAT contributed to the study design and data collection. All authors read and approved the final manuscript.

## Pre-publication history

The pre-publication history for this paper can be accessed here:

http://www.biomedcentral.com/1471-2458/12/27/prepub
